# Fundamental motifs and parity within the crystallographic point groups

**DOI:** 10.1107/S1600576725005631

**Published:** 2025-07-29

**Authors:** Maureen M. Julian, Matthew Macauley

**Affiliations:** a Department of Materials Science and Engineering Virginia Tech Blacksburg VA USA; b School of Mathematical and Statistical Sciences Clemson University Clemson SC USA; Wilfrid Laurier University, Waterloo, Ontario, Canada

**Keywords:** crystallographic point groups, abstract groups, crystal systems, family trees, Hasse diagrams, motifs, subgroups, parity

## Abstract

This paper analyzes the Hasse diagram, or family tree, of the 3D crystal classes, also called geometric crystal classes. The 32 point-group classes are partitioned into seven crystal systems. In this paper, the structures of these systems are analyzed, leading to a new understanding of the relationships among and within them. The point groups, including their subgroups up to conjugacy, appear in six structural *motifs* in the Hasse diagram or family tree. Each motif has a *parity* – even or odd – that determines its structure. In three dimensions, the odd motifs are called monads, trigonals and cubics, and the even motifs are called dyads, tetragonals and hexagonals. Of the 32 classes of 3D point groups, 29 have a well defined parity, in that they appear in either an even or an odd motif. In contrast, the three monoclinic point groups are ‘ambidextrous’, in that they appear in two motifs, one of each parity. An analysis of the ten 2D point groups reveals an analogous structure, except for the presence of an ambidextrous crystal system. The striking structural uniformity of the motifs across the Hasse diagram confirms that they are essential building blocks of the crystallographic point groups.

## Abstract 2

The Hasse diagram of the 3D point-group classes is built with six motifs that have a well defined parity that determines their structure. Of the seven crystal systems, three are built with odd motifs, three are built with even motifs and the last one, the monoclinics, is ‘ambidextrous’ as they are built with both.

## 1. Introduction

Table 2.1.1.1 in *International tables for crystallography*, Volume A, *Space-group symmetry* (2016 ▸) (hereafter denoted as *ITA*) partitions the 32 three-dimensional crystal classes^1^ into seven crystal systems: triclinic, monoclinic, orthorhombic, tetragonal, trigonal, hexagonal and cubic. The subgroup relationships among these groups define a *partially ordered set*, or *poset*, which can be visualized by a graph called its *Hasse diagram*,^2^ where edges (lines) represent minimal relationships. The Hasse diagram of the point groups can be found in *ITA* (pp. 731–732), as well as in textbooks such as *Foundations of crystallography* (Julian, 2015 ▸, Chapters 3 and 4). The crystal system classification used in *ITA* is driven by the corresponding Bravais lattices. There are 14 such lattices, and Hasse diagrams have also been used in their analysis (Flack, 2015 ▸).

This paper presents a new perspective on the point-group Hasse diagram by introducing six structural motifs from which it is built. Each motif has a well defined parity that determines its structure: three are even, and three are odd. Of the seven crystal systems, three consist of groups that are only found in even motifs, and three of them appear exclusively in odd motifs. The groups in the last crystal system, the monoclinics, appear in both motifs. The analysis of these motifs, and the abstract groups within and across them, is done throughout Section 2. The 32 point groups fall into 18 isomorphism types, involving direct products of cyclic groups *C*_*n*_, dihedral groups *D*_*n*_,^3^ symmetric groups *S*_*n*_ and alternating groups *A*_*n*_.^4^ There is a striking regularity among the abstract groups across motifs if care is taken when writing them consistently, especially in the degenerate cases, *e.g.A*_3_ ≅ *C*_3_, *S*_2_ ≅ *C*_2_ and *A*_2_ ≅ *C*_1_. For example, patterns may only emerge upon writing the cyclic group *C*_6_ as *S*_2_ × *C*_3_ or *A*_3_ × *C*_2_, or the cyclic group *C*_2_ as *A*_2_ × *C*_2_. Some abstract groups appear in multiple motifs, and of both parities. In other words, the isomorphism type is not an invariant of the crystal system, the motif or even the parity. At times in this paper, the usual names of the point groups are adjusted to emphasize the aforementioned parallelisms. In Section 3, an analogous exercise is done for the Hasse diagram of the ten 2D point groups, and similar structural motifs with an explicit parity are found. The paper concludes in Section 4 with a summary and comparison of the motifs and the groups that contain them, by both their crystal systems and their abstract isomorphism types.

Though the partitions of the point groups into crystal systems and motifs are different, they are complementary rather than contradictory and are both very natural. When viewed together, they provide fresh insights into the structure of the point groups, and new ideas, such as the parities and alternative group names. This should be of general interest to crystallographers, especially among students, educators and those inspired by the more theoretical aspects of symmetry in crystallography.

## 2. Three-dimensional point-group motifs

### 2.1. The 32 groups and their Hasse diagram

**Figure 1:**
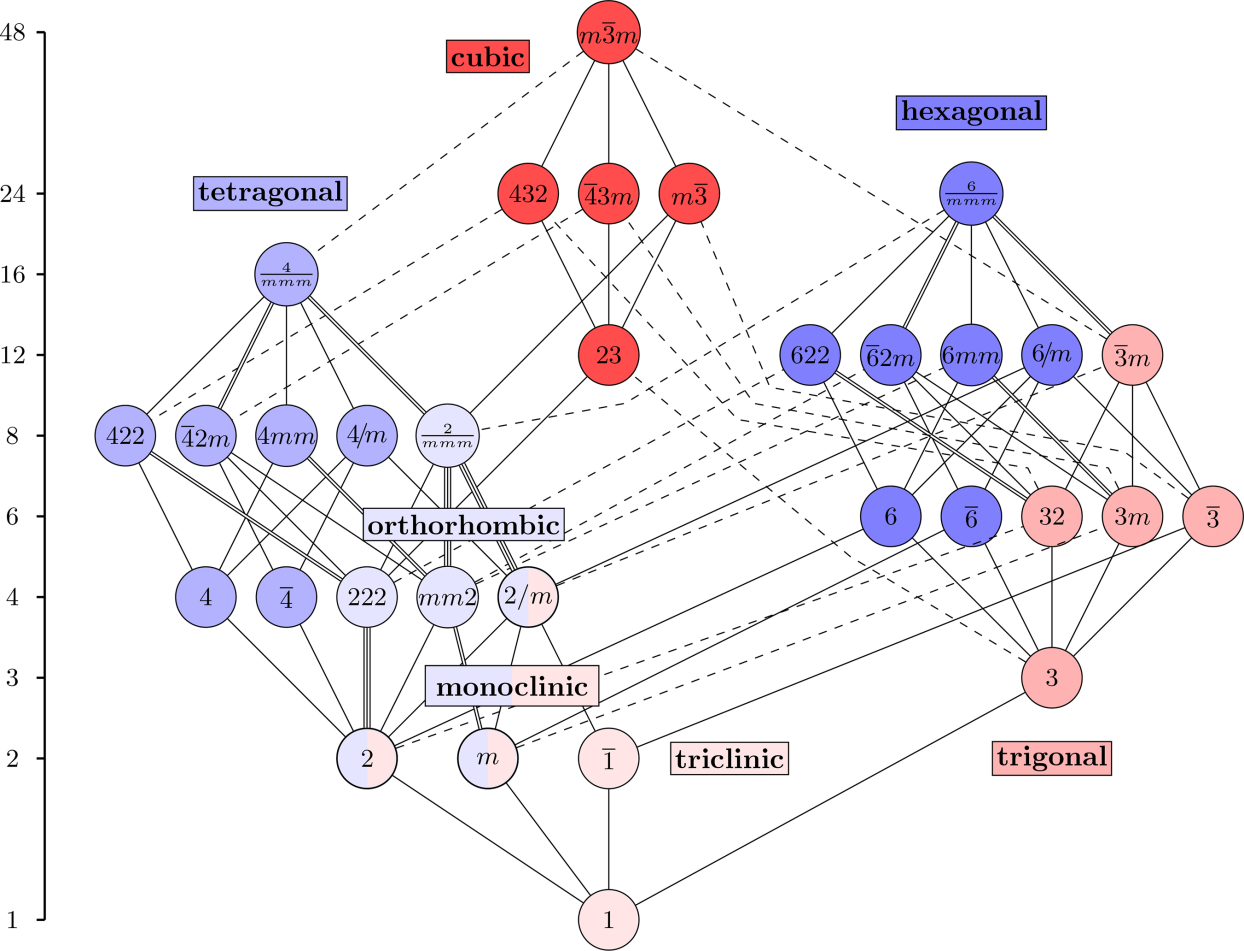
The Hasse diagram of the 32 3D crystallographic point groups in the Hermann–Mauguin notation, with the order of the groups shown on the left.

Formally, a poset is a set *X* with a binary relation ≤ that is reflexive, antisymmetric and transitive. Its Hasse diagram is the graph with the elements of the poset as its nodes, and *x* and *y* are connected by an edge if one of them *covers* the other. Recall that *y* covers *x* if *x* ≤ *y* and *x* ≤ *z* ≤ *y* implies *z* = *x* or *z* = *y*. The 32 three-dimensional crystallographic point groups form a poset, where 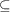 is the relation. The Hasse diagram of this poset is thus a graphical representation of the maximal subgroups and minimal supergroups, and it appears in Fig. 1 ▸. One group arises as a subgroup of another if there is a rising path between them. In other words, reading the diagram upwards reveals minimal supergroups, while reading it downwards reveals maximal subgroups. This diagram contains more information than just the poset structure. Solid lines represent maximal normal subgroups and dashed lines represent non-normal subgroups, *i.e.* multiple conjugate subgroups. The double and triple solid lines denote the presence of two or three distinct non-conjugate (normal) subgroups, respectively. This Hasse diagram, without the colors, is related to Fig. 3.2.1.3 and Table 2.1.1.1 of *ITA*. The six structural motifs introduced in this paper are denoted by color and shading. The three even motifs are distinguished by different shades of blue and the three odd motifs by different shades of red. The three groups that appear in multiple motifs are bi-colored.

### 2.2. Extraction of the crystal systems from the Hasse diagram

**Figure 2:**
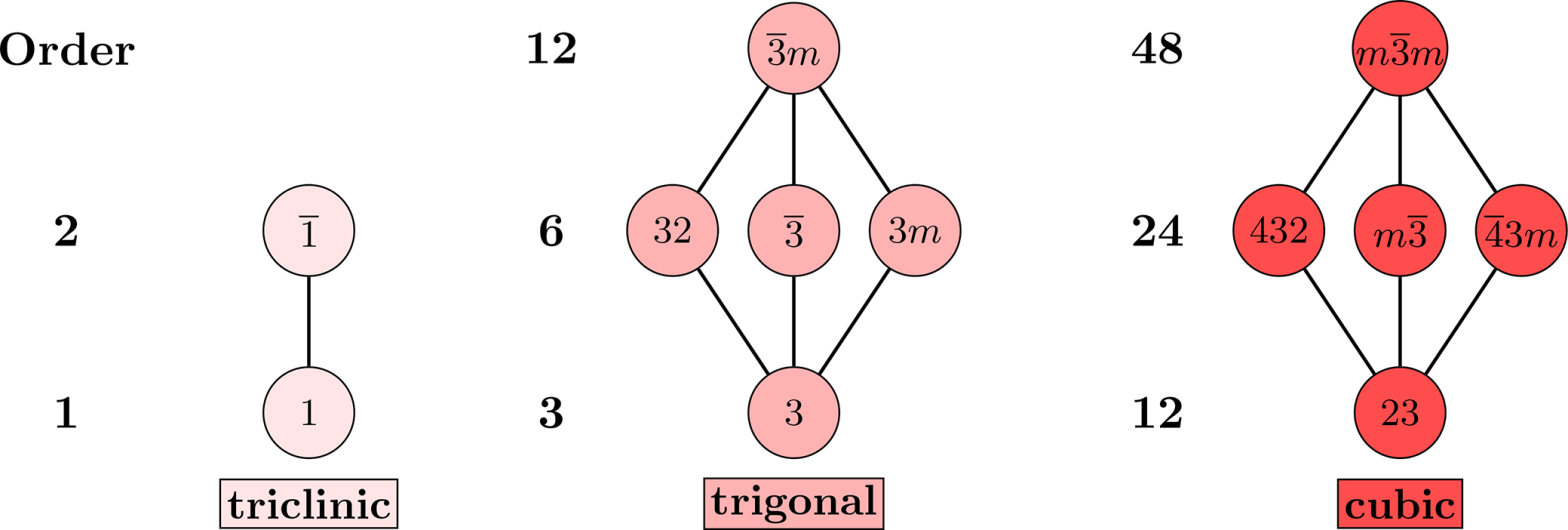
Triclinic, trigonal and cubic crystal systems extracted from the Hasse diagram.

**Figure 3:**
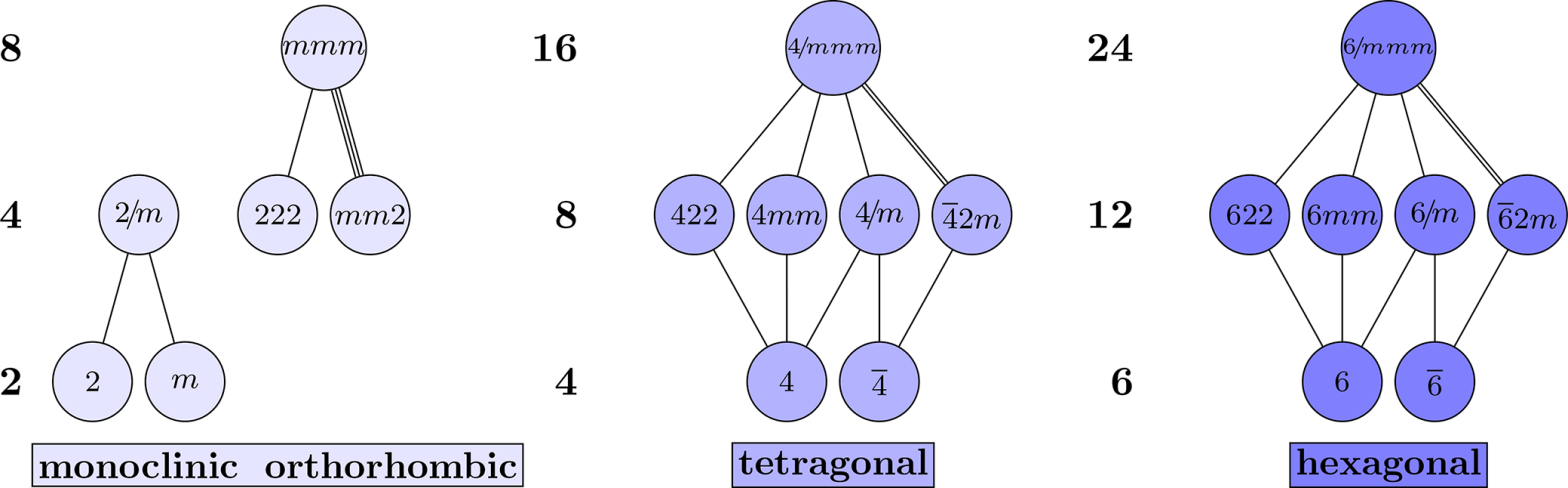
Monoclinic, orthorhombic, tetragonal and hexagonal crystal systems extracted from the Hasse diagram.

The first step in identifying the motifs defined in this paper is to extract the seven crystal systems from Fig. 1 ▸. This is done by first identifying the seven holohedry groups, or lattice point groups: 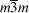, 6/*mmm*, 4/*mmm*, 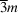, *mmm*, 2/*m* and 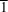. For each of these, its crystal system consists of it with all subgroups in the Hasse diagram that are not contained in a smaller holohedry. The result is shown in Figs. 2 ▸ and 3 ▸. Notice the similarity of structure between the trigonals and the cubics in Fig. 2 ▸ and, separately, between the tetragonals and the hexagonals in Fig. 3 ▸. These parallels can also be seen between the Hermann–Mauguin names. Table 1 ▸ summarizes the information from *ITA*. Since subgroups of index 2 are normal, all edges within any crystal system must be solid.

In contrast, the triclinic system does not resemble the other two classes in Fig. 2 ▸, nor it is apparent that it even should – one might say that this crystal system is a ‘degenerate’ version of the others, due to it just being the Hasse diagram of a cyclic group of order two. A similar observation can be made in Fig. 3 ▸. Here, the groups in the monoclinic and orthorhombic systems^5^ have analogs in the tetragonal and hexagonals, but there still seems to be something missing. As will be shown in this paper, these apparent degeneracies are artifacts of the crystal system designation that derives from a Bravais lattice type viewpoint. That is not to say that they are wrong, but rather they are just one piece of the whole picture. The triclinics naturally join with some of the monoclinics to form a new motif that has the same structure as the trigonal and cubics, from Fig. 2 ▸, and the point-group Hasse diagram is built from precisely these motifs, though several will appear more than once. This inspires the new names of these motifs: the *monads* and *dyads*, respectively. At this point, it should not even be clear how or why such a structural uniformity exists.

### 2.3. Odd motifs: monads, trigonals and cubics

**Figure 4:**
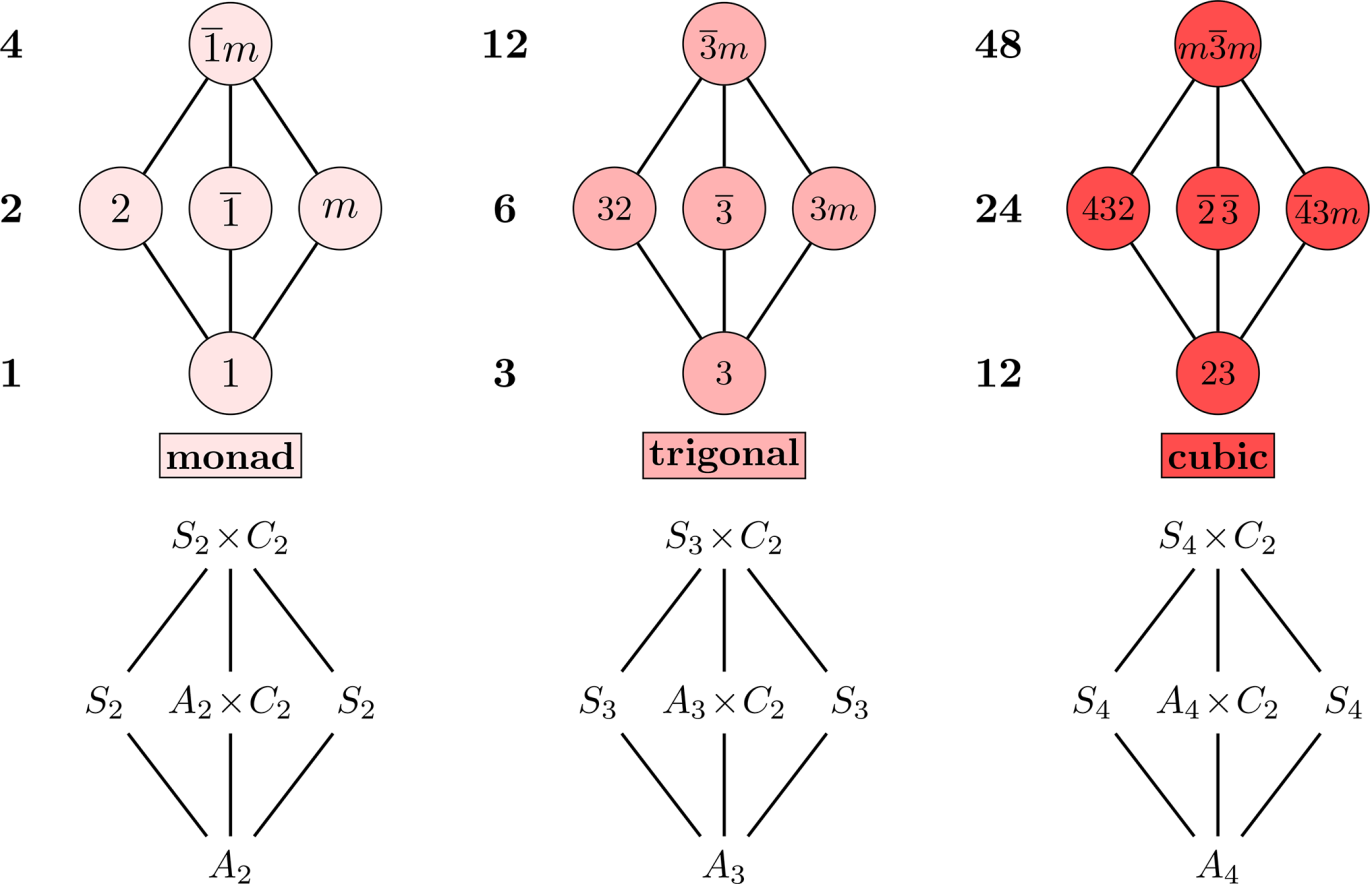
The three odd diamond-shaped motifs in both Hermann–Mauguin and abstract algebra notations. Note that *A*_2_ × *C*_2_ ≅ *C*_2_ and *A*_3_ × *C*_2_ ≅ *C*_6_.

The three odd motifs are diamond-shaped, with a single maximal group and minimal subgroup. The quotient of these two groups is the Klein four-group, *C*_2_ × *C*_2_ ≅ *D*_2_, which has a distinctive diamond-shaped Hasse diagram. These motifs appear in the point-group Hasse diagram because *D*_2_ is a *subquotient* of the maximal cubic group 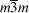. All three odd motifs are shown in Fig. 4 ▸. The cubics and trigonals consist of precisely the groups from the crystal system of the same name. The cubics can be characterized by a minimum of two nonparallel 3 or 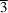 axes and the trigonals by a single 3 or 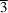 axis. The final odd motif, called the *monads*, consists of triclinic and monoclinic point groups. Just as the maximal trigonal group, 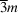, contains 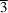 and 3*m*, the maximal monad group, 2/*m*, contains 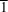 and *m*. This explains the non-standard naming of 2/*m* as 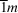 in Fig. 4 ▸. Another creative name, meant to highlight the analogous structure of these motifs, is the renaming of 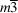 as 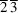. The minimum subgroups of the monads, trigonals and cubics are point groups 1, 3 and 23 with respective orders of 1, 3 and 12. Note also that each of these motifs contains point groups 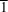, 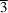 and 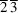 (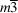) with respective orders of 2, 6 and 24. Thus, in general, if the minimum subgroups are *g* = 1, 3, 23 with order *n*, then 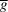 has order 2*n*.

At the bottom of Fig. 4 ▸ are the abstract groups: *S*_*n*_ is the symmetric group of order *n*!, *A*_*n*_ is the alternating group of order *n*!/2 and *C*_*n*_ is the cyclic group of order *n*. Though some of the abstract group labels are unorthodox, *e.g.A*_2_ is the trivial group and *S*_3_ × *C*_2_ is the dihedral group *D*_6_ of order 12, these are carefully chosen to highlight the similarities between the motifs. For another example, the group 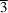 is classically written as *C*_6_, but that is isomorphic to *C*_3_ × *C*_2_, and the alternating group *A*_3_ is *C*_3_. The uniformity of the abstract groups across the odd motifs provides evidence to the claim that these motifs are essential building blocks of the Hasse diagram.

For another example of this uniformity, in terms of the abstract groups, 3*m* ≅ *S*_3_ in the trigonals is the symmetry group of a triangle, which can be realized as the convex hull of the unit basis vectors *e*_1_ = (1, 0, 0), *e*_2_ = (0, 1, 0) and *e*_3_ = (0, 0, 1) in 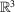. Going up one dimension, the convex hull of *e*_1_, …, *e*_4_ in 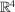 is a tetrahedron, whose symmetry group is 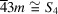 in the cubic motif. Going down one dimension, the convex hull of *e*_1_ = (1, 0) and *e*_2_ = (0, 1) in 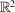 is a line segment – a ‘one-dimensional triangle’. Its symmetry group is *m* ≅ *S*_2_ ≅ *C*_2_, the corresponding point group in the monad motif. In four dimensions, the crystal system contains more than just the groups between *S*_5_ × *C*_2_ and *A*_5_ in the Hasse diagram, *e.g.* the Frobenius group *F* (an affine general linear group) of order 20 and *F* × *C*_2_ (Hurley, 1951 ▸; Mozrzymas & Solecki, 1975 ▸).

**Figure 5:**
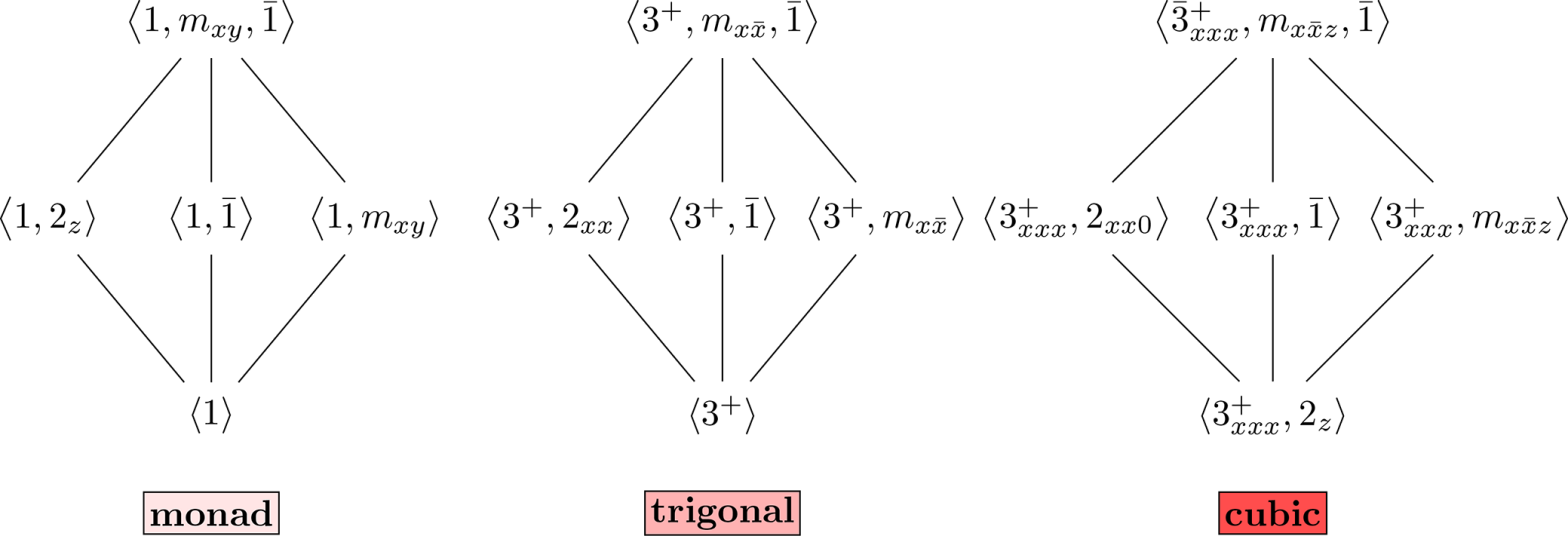
Generators for the point groups in the odd motifs.

More parallels can be seen between the point groups in the odd motifs by examining the specific generators that correspond to each group. Though there are many choices of generating sets, particular ones can be chosen to match the abstract group names given in Fig. 4 ▸ and the uniformity described in the previous paragraph. These appear in Fig. 5 ▸. Nomenclature for the symmetry operations (generators) is taken from Appendices 2 and 3 in the book by Julian (2015 ▸). The maximal group is 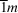 (2/*m*), which has four symmetry operations: 1, 2_*z*_, *m*_*xy*_ and 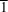. To facilitate the parallelism with the other odd motifs, consider this group generated (non-minimally) by 1, *m*_*xy*_ and 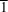. The maximum group of the trigonals may be generated (non-minimally) by the threefold 3^+^, 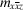 and 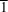. Finally, the maximal *cubic* group is generated, also not minimally, by a threefold rotoinversion 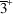 along a diagonal, 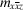 and 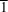.

### 2.4. Even motifs: dyads, tetragonals and hexagonals

**Figure 6:**
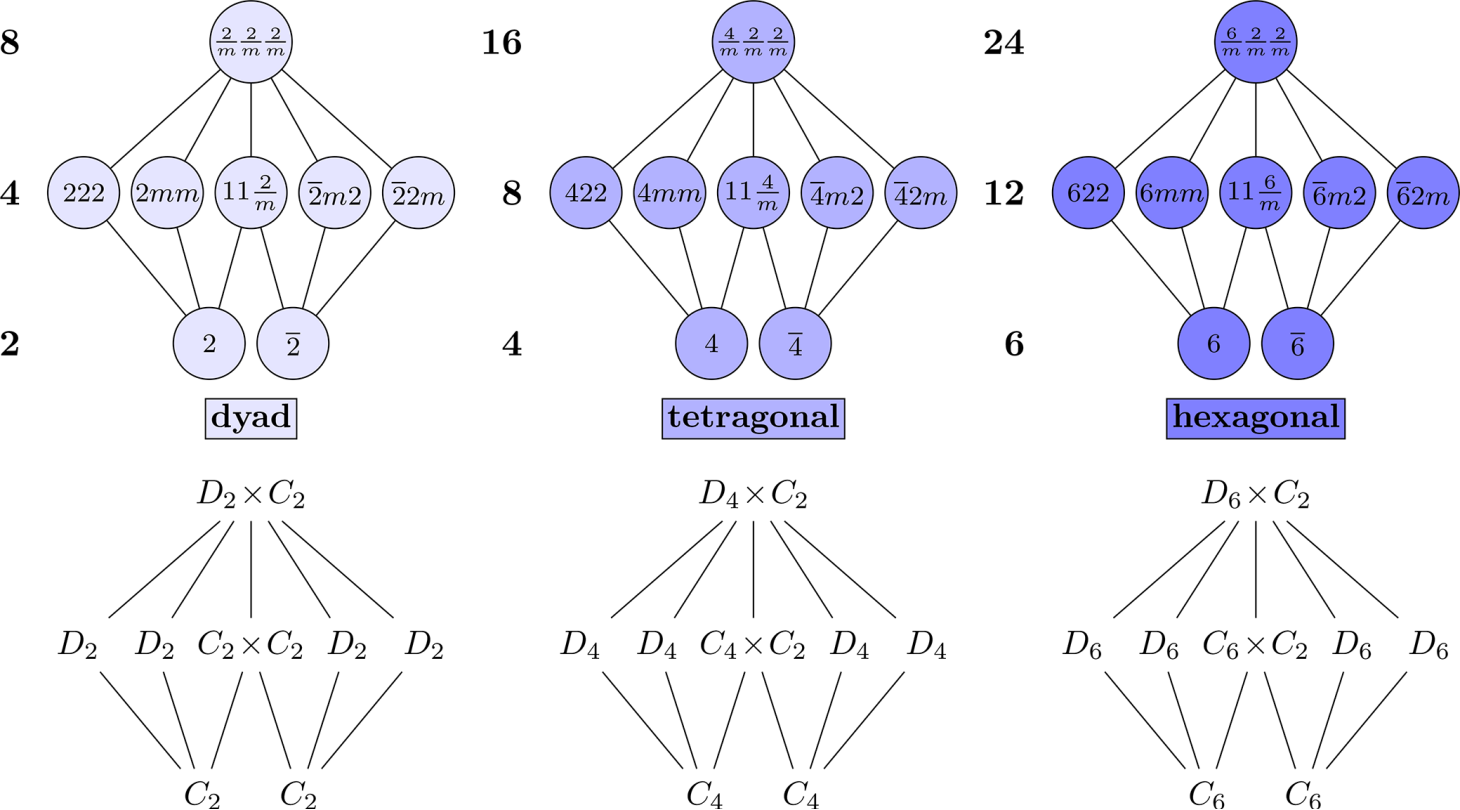
The three even double-diamond motifs in both Hermann–Mauguin and abstract algebra notations.

The three even motifs are characterized by their maximal subgroup *n*/*mmm*, which is the direct product *D*_*n*_ × *C*_2_ of a dihedral group. They also have two minimal subgroups, *n* or 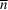, which are both the cyclic group *C*_*n*_. Since the quotients of *n*/*mmm* with *n* and 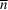 are the Klein four-group, the even motifs have a ‘double diamond’ or ‘double Klein’ shape; see Fig. 6 ▸. Two of these motifs, the hexagonals and tetragonals, are precisely the groups in the corresponding crystal system of the same name. At this point, it should be less clear how the dyad motif arises, because it consists of groups from the monoclininc and orthorhombic crystal systems.

Fig. 6 ▸ shows the result of ‘pulling apart’ the tetragonal and the hexagonal motifs via the double line in Fig. 1 ▸, as described earlier. For example, the tetragonal point group 4/*mmm* of order 16 has two normal index-2 subgroups of type 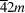. Crystallographically, these are related by a rotation of 45°, so they are distinguished as 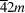 and 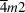, respectively. As shown in Table 2 ▸, the symmetry operations 1, 2_*z*_, 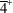 and 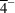 are common to all three of these point groups; symmetry operations 4^+^, 4^−^, 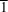 and *m*_*xy*_ occur only in 4/*mmm*; symmetry operations 2_*x*_, 2_*y*_, 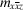 and *m*_*xxz*_ occur only in 4/*mmm* and 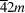; and 2_*xx*_, 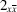, *m*_*xz*_ and *m*_*yz*_ occur only in 4/*mmm* and 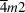. A similar argument can be made for the hexagonal point group 6/*mmm* of order 24, and subgroups 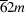 and 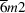, each of order 12. Finally, the dyad 2/*mmm* (or *mmm*) can be similarly arranged where 2/*mmm* parallels 4/*mmm* and 6/*mmm*, and 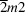 and 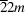 parallel 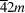 and 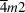
*etc*. Note the non-standard use of 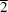 = *m* to facilitate the parallelism. The two minimal subgroups of the dyads, tetragonals and hexagonals are 2 and 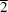, 4 and 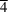, and 6 and 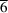, with respective orders of 2, 4 and 6. In contrast to the odd motifs, the minimal subgroups in all three even motifs are *g* = *n* and 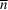, which both have order *n*.

### 2.5. Motif parity

The distinction into odd and even motifs can be motivated by the parity of a rotation that each maximal group contains. The rotoinversion (which generates the group 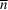) has order 2*n* if *n* is odd. This is twice the order of the corresponding rotation. The odd motifs can be described in a uniform way by their maximal groups, 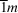, 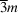 and 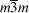. Each contains a planar subgroup 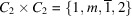, and is generated by this and an additional rotation of odd order. For the monad this is the trivial onefold rotation, for the trigonal it is a threefold rotation with axis perpendicular to the plane of the subgroup, and for the cubic there are threefold rotations with axes along the diagonals of the cube.

If *n* is even, then the rotoinversion that generates the group 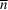 has order *n*, the same as the corresponding rotation, and the group 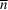 does not contain an inversion point. This leads to each even motif having two minimal subgroups. The even motifs can also be described systematically by their maximal groups, *mmm*, 4/*mmm* and 6/*mmm*. Each of these is generated by a planar subgroup *C*_2_ × *C*_2_ and a single additional rotation along an axis perpendicular to this plane. For the dyad this is a twofold axis, for the tetragonal it is a fourfold axis, and for the hexagonal it is a sixfold axis. The parity of the additional rotation in this maximal group characterizes the parity of the motif, which motivates our names of ‘even’ and ‘odd’.

### 2.6. Dyads, monads and the ambidextrous point groups

Most of the 32 point groups appear only in an even motif or only in an odd motif. For convenience, these point groups will occasionally be referred to as ‘even’ or ‘odd’. However, several different isomorphic point groups appear in motifs of opposite parity. Thus, the parity is a property not of the abstract groups but of the context in which they occur. As will be shown in this section, three point groups appear in both even and odd motifs, and these are called *ambidextrous*.

**Figure 7:**
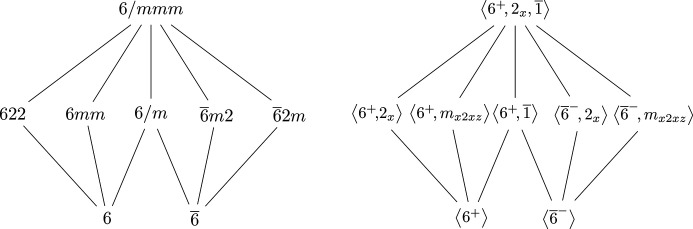
The Hasse diagram of the hexagonal point group 6/*mmm* with the double lines (pairs of normal subgroups of the same point-group type) pulled apart.

The similarity of the abstract groups across the even motifs is arguably more apparent than it is for the odd motifs; the only non-standard convention needed is to write *D*_2_ × *C*_2_ ≅ *C*_2_ × *C*_2_ × *C*_2_. On the other hand, analyzing the subgroup structure of *mmm* (henceforth 2/*mmm*) is more complicated than any of the odd motifs. Before getting there, the hexagonal and tetragonal motifs must be analyzed first. Taking the hexagonals as an example, the rotation can be chosen as 6^+^, the twofold rotation can be taken to be 2_*x*_, and the inversion is 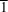. Generators are shown in Fig. 7 ▸. The tetragonal motif is completely analogous: just with 4 in place of 6.

**Figure 8:**
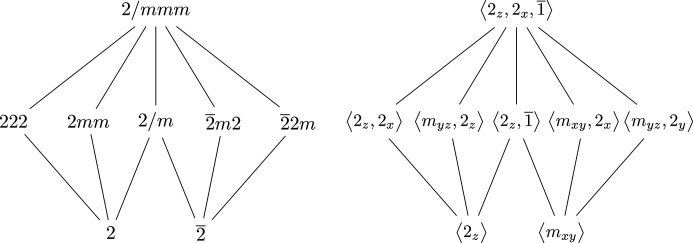
The Hasse diagram of the dyads, the *n* = 2 analog of the hexagonals; compare with Fig. 7 ▸.

The even motifs shown in Fig. 7 ▸ are enough to construct an analogous picture for the dyads. The difficulty here is that the *n*-fold rotation, for *n* = 2, commutes with the twofold rotation, and so the resulting group, *D*_2_ × *C*_2_, is abelian. This leads to some of the monoclinic point groups being assigned to odd motifs, whereas the remaining ones are part of the dyads. To distinguish the subgroups, the sixfold rotation 6^+^ in 6/*mmm* is replaced with 2_*z*_. It is orientation preserving (positive determinant) and hence a rotation. The dyad motif, in terms of both the point groups and their generators, is shown in Fig. 8 ▸. It should be immediately apparent how the Hasse diagram in Fig. 8 ▸ is analogous to both the tetragonal and the hexagonal motif.

**Figure 9:**
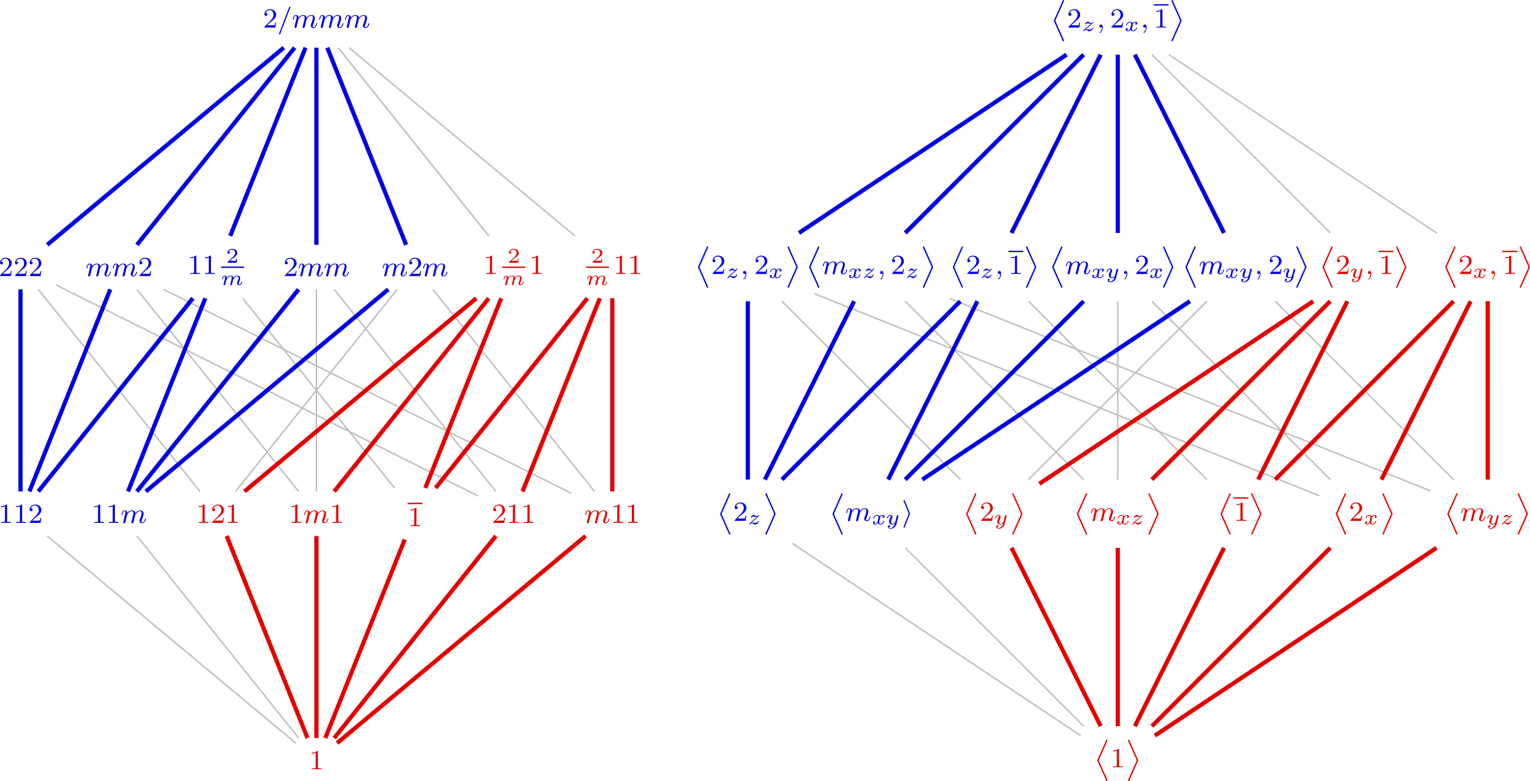
Upon fixing a primary axis, the subgroups of 2/*mmm* break down into one (even) dyad motif and two (odd) monad motifs, that intersect in the triclinic crystal system, 1 and 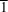.

The maximal dyad point group 2/*mmm*, which is isomorphic to *D*_2_ × *C*_2_, has 16 subgroups: the group itself, seven noncyclic groups of order 4, seven groups of order 2 and one group of order 1. However, if the dyads are to have the same structure as the hexagonals, the motif should only have five order-4 subgroups and two order-2 subgroups. The next step is to understand how the dyad motif embeds into the Hasse diagram of the subgroups of 2/*mmm*, which is shown in Fig. 9 ▸. The dyads are colored blue. Of the remaining two groups of order 4, note that both are in a monad motif. These are 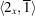 and 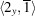, and they are colored red to highlight their structure. These two monad systems intersect in precisely the triclinic crystal system: 1 and 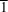.

Once a primary axis is chosen, then one of the three *m* point groups joins the (even) dyads. On the other hand, the other two *m* groups are part of the (odd) triclinics. Similarly, the particular point group 2 (generated by 2_*x*_, 2_*y*_ or 2_*z*_) is also determined by the choice of primary axis and is part of the dyads. The other two groups become part of the triclinics. The groups that have this ‘ambidextrous’ property, which also include the order-4 groups generated by the aforementioned elements, are the three monoclinics. In contrast, the ortho­rhombic groups appear in a dyad motif, whereas the triclinics are in a monad motif.

### 2.7. Fused odd motifs

**Figure 10:**
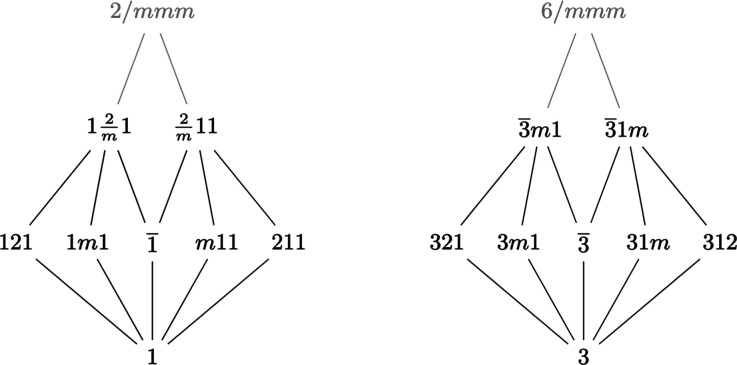
The monad and trigonal motifs come in pairs that share common point groups.

As shown in Fig. 9 ▸, the maximal dyad group 2/*mmm* and its 16 subgroups contain one dyad motif, and two monad motifs, that share its triclinic subgroups (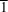 and 1). Together, these fused monad motifs make an ‘upside-down even’ motif in the Hasse diagram, as shown in Fig. 10 ▸ on the left.

There is another fused monad pair in the 3D point-group Hasse diagram, but it is hidden by the double and dashed lines that represent multiple subgroups. The maximal hexagonal group 6/*mmm* contains two normal 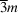 subgroups, as indicated by the solid double line. In other words, the Hasse diagram of 6/*mmm* and its subgroups contains two copies of an odd motif, which share their common 3 and 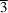 subgroups.

Going back to the Hasse diagram in Fig. 1 ▸, note that the maximal cubic subgroup 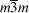 contains a dashed line to the maximal trigonal subgroup, which has index 4 (order 12). Therefore, 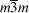 contains *four* conjugate maximal trigonal subgroups,^6^ which arise as two pairs of fused trigonals, as in Fig. 10 ▸.

The six motifs that appear in the 3D point-group Hasse diagram, along with the groups they contain, are summarized in Table 3 ▸. The Hermann–Mauguin notation appears in the table at the top, and the abstract groups are below. Recall that several of these are given non-standard names to emphasize their analogs in the other motifs of the same parity, *e.g.*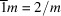, 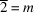, 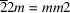 and 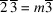. Additionally, several abstract groups are written non-traditionally, *e.g.A*_2_ × *C*_2_ ≅ *C*_2_ and *S*_2_ × *C*_2_ ≅ *D*_2_ × *C*_2_ ≅ *C*_2_ × *C*_2_, to highlight the regularity across motifs. Note that there are multiple abstract groups that arise in more than one crystal system and motif, and specifically four that appear in motifs of opposite parity. These are the dihedral groups *D*_6_ and *D*_2_, and the cyclic groups *C*_6_ and *C*_2_.

## 3. Two-dimensional point-group motifs

Following the analysis of the 3D crystallographic point groups, it is natural to revisit the ten 2D groups, which form a subset of the 3D groups. In *ITA*, the seven 3D crystal systems reduce down to four 2D systems: the cubics have no analog, the trigonals join the hexagonals, the tetragonals are renamed the squares, and the remaining groups, which come from the triclinics and monoclinics, become the obliques. This classification is summarized in Table 4 ▸(*a*).

**Figure 11:**
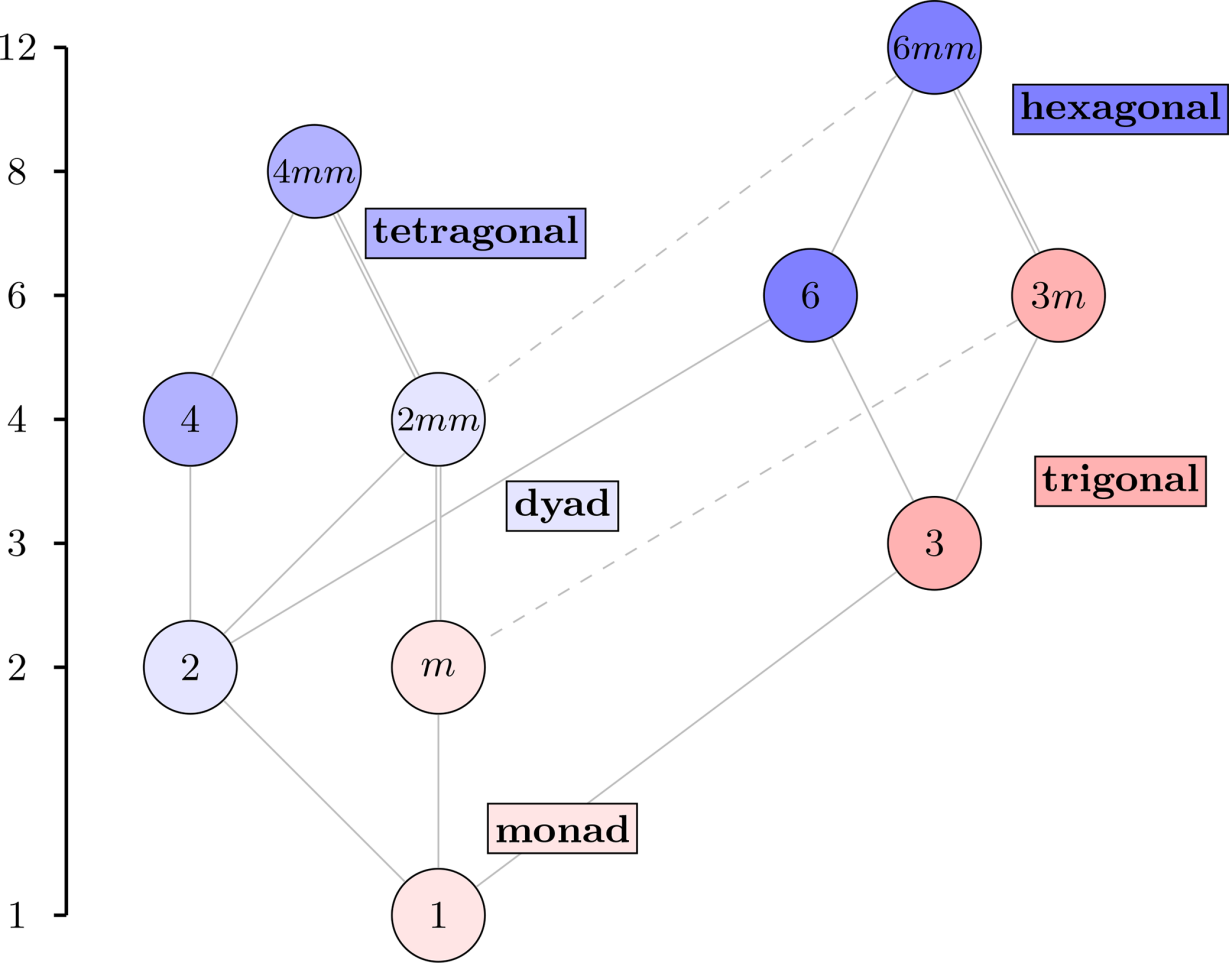
The Hasse diagram of the ten 2D crystallographic point groups, with the order of the groups shown on the left.

Much like in the 3D case, the Hasse diagram of the 2D point groups is also built from regular motifs of either even or odd parity. Each cyclic point group *n*, for *n* = 1, 2, 3, 4, 6, is paired with the corresponding dihedral point group, which is *nm* if *n* is odd and *nmm* if *n* is even. This is summarized in Table 4 ▸(*b*). The soundness of the parity-based classification of this paper is even more apparent with the 2D groups. Fig. 11 ▸ shows the Hasse diagram of the 2D point groups, as it appears in *ITA*, but annotated with the five motifs.

**Figure 12:**
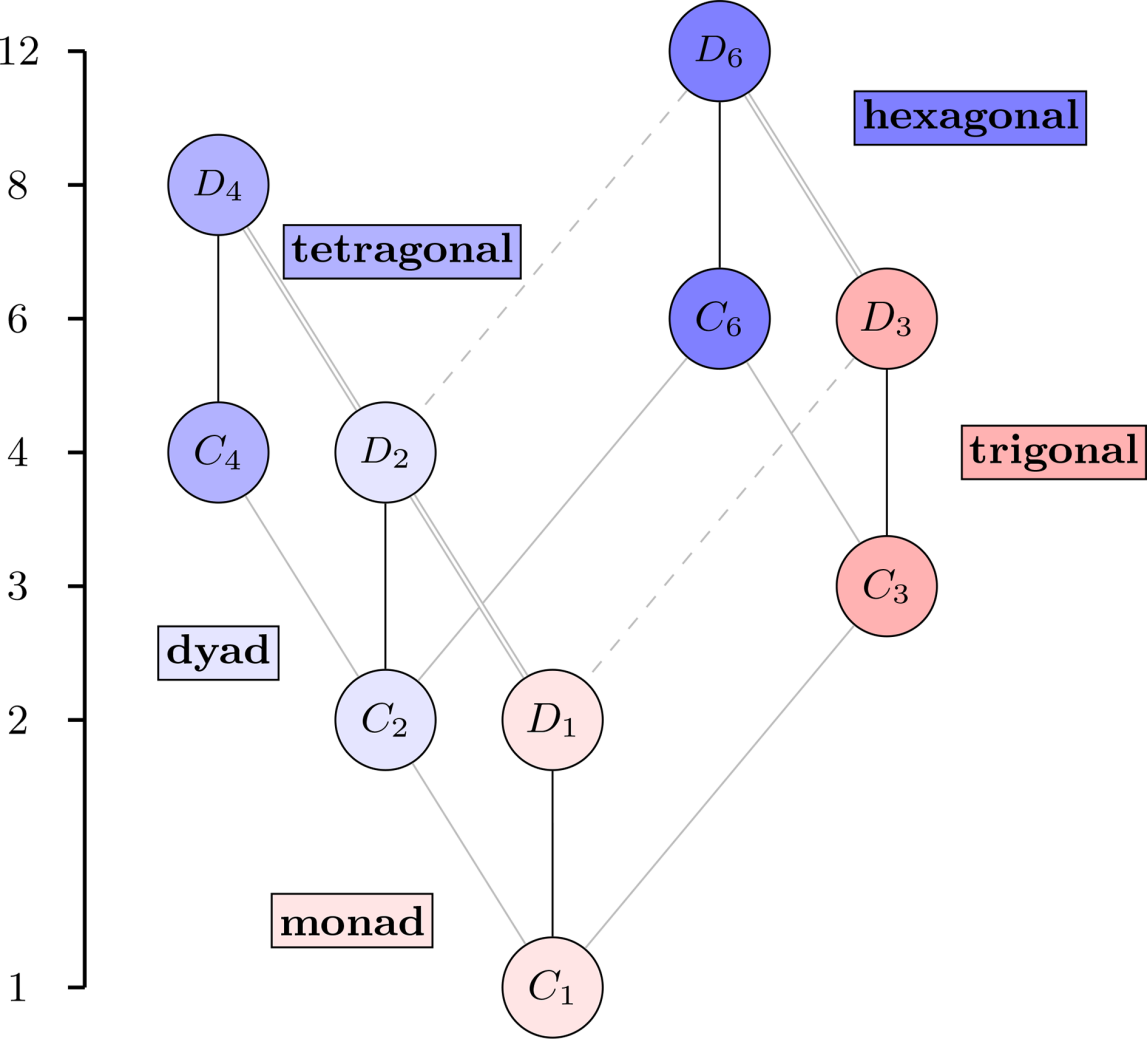
The Hasse diagram of the ten 2D crystallographic point groups, labeled by the abstract groups.

Not only does each class in Fig. 11 ▸ consist of the cyclic group of rotations, denoted *n*, and the corresponding dihedral group (*nm* or *nmm*), but the parity agrees with the number of *m*’s in the dihedral group. The isomorphism types of these groups are shown in Fig. 12 ▸. This time, the Hasse diagram is rearranged from how it appears in *ITA* to highlight the uniformity and the motif structure, which is not present in Fig. 11 ▸. The fact that there are no ambidextrous 2D point groups makes sense, considering that those only arose upon picking a particular axis in 3D space.

## 4. Concluding remarks

One of the overarching themes of this paper is the parity of the motifs from which the point-group Hasse diagrams are built and how the crystal classes fit into this framework. This parity is not an invariant of the abstract group type. Indeed, of the 18 abstract groups that arise as 3D point groups, four of them appear in both even and odd motifs. Table 5 ▸ provides a summary of this, by listing all point groups and the parity of the motif(s) in which they appear.

**Figure 13:**
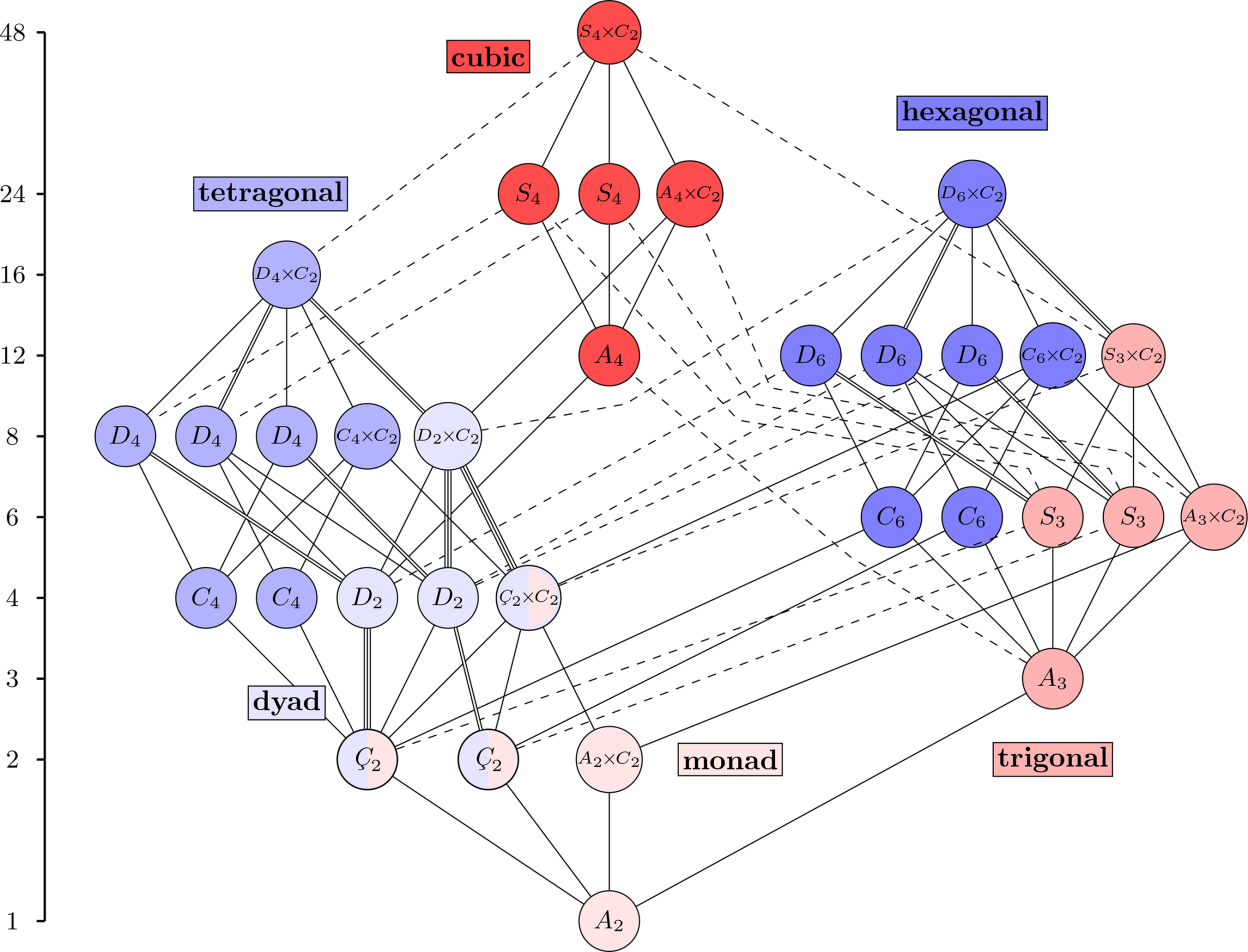
The Hasse diagram of the 3D crystallographic point groups in abstract notation, with the order of the groups shown on the left. The ambidextrous groups are bicolored, and *Ç*_2_ denotes the fact that *S*_2_ was used in the dyads and *C*_2_ in the monads. Boxes indicate isomorphic crystal point groups. Fig. 1 ▸ and Table 3 ▸ support Fig. 13 ▸.

It is also insightful to see the abstract groups not just in a table but back on the Hasse diagram. This is shown in Fig. 13 ▸, with the same coloring by motifs as was done earlier. The three ambidextrous groups (the monoclinics: 2*mm*, *m* and 2/*m*) appear in the bicolored nodes. Up to this point, when part of the (odd) monads, their abstract groups have been written as *S*_2_ × *C*_2_, *S*_2_ and *S*_2_, to match the pattern of the corresponding groups in the cubics and trigonals. However, when in the (even) dyads, they have been written as *C*_2_ × *C*_2_, *C*_2_ and *C*_2_. Naturally, this difference is only cosmetic, because *S*_2_ ≅ *C*_2_. Rather than pick either *C*_2_ or *S*_2_ to write in each bicolored node in Fig. 13 ▸, the French cedilla – a ‘soft *C*’ – is used to write the group as *Ç*_2_, playfully emphasizing the difference in notation depending on which motif it appears in.

In closing, one of the important takeaways from this paper is that, though the partitions of the point groups by crystal systems and by the motifs introduced here are different, they are complementary, and they add value when viewed together. For one example, since the crystal system classification is driven by the Bravais lattices, the hexagonal and trigonal point groups are closely related, because both hexagonal Bravais lattice unit cells have *a* = *b* and α = β = 90°, γ = 120°. The Bravais lattice types are intrinsically periodic, and the periodicity of the crystallographic point groups is achieved by allowing symmetries permitted in the periodic lattice. In this paper, the hexagonal and trigonals, as motifs, have opposite parity. Both of these viewpoints are insightful, and they are equally valid. The structural uniformity of the even and odd motifs, and how they fit together in the Hasse diagram, validates the idea that they are essential building blocks of the crystallographic point groups. This is further supported by the 2D points groups, which are a subset of the 3D point groups, and whose Hasse diagram is also built with even and odd motifs, albeit simpler ones. It would be interesting to see if analogous patterns arise in higher-dimensional point groups. Finally, from an educational standpoint, it can be illuminating to see how the motif structure suggests that certain point groups could be given alternative names. For example, in Hermann–Mauguin notation, the largest monoclinic group 2/*m* can be thought of as 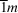 when it appears in an odd motif. One can indeed justify referring to the orthorhombic group *mm*2 as 2*mm* due to its odd motif analogs, 4*mm* and 6*mm*. Similarly, the cubic group 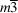 can be thought of as 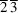, to parallel 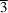 in the trigonals and 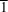 in the monads. Though the authors are not advocating for a change in these well established names, it is insightful to see these alternative aliases, and other aspects of the point groups that the motif structure brings to light, especially when in synergy with the crystal systems.

## Figures and Tables

**Table 1:** Crystal systems for 3D point groups from *ITA* Table 2 ▸.1.1.1

Crystal system		Crystallographic groups, Hermann–Mauguin notation						
Triclinic		1	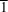					
Monoclinic		2	*m*	2/*m*				
Orthorhombic					222	*mm*2		*mmm*
Tetragonal		4	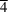	4/*m*	422	4*mm*	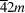	4/*mmm*
Hexagonal	*Trigonal*	3	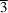		32	3*m*	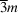	
	*Hexagonal*	6	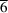	6/*m*	622	6*mm*	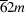	6/*mmm*
Cubic		23	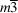		432	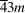	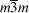	

**Table 2:** Comparison of symmetry operations of point groups 4/*mmm* (order 16) with subgroups 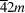 and 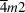 (order 8)

Group	Symmetry operations															
4/*mmm*	1	2_*z*_	4^+^	4^−^	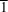	*m*_*xy*_	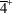	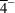	2_*y*_	2_*x*_	2_*xx*_	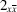	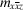	*m*_*xxz*_	*m*_*xz*_	*m*_*yz*_
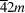	1	2_*z*_					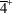	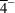	2_*y*_	2_*x*_			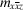	*m*_*xxz*_		
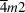	1	2_*z*_					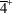	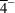			2_*xx*_	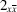			*m*_*xz*_	*m*_*yz*_

**Table:** 

Parity	Motif	Point groups, Hermann–Mauguin notation						
Odd	Monad	1	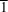		2	*m*	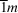	
	Trigonal	3	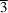		32	3*m*	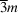	
	Cubic	23	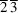		432	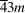	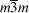	

Even	Dyad	2	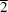	2/*m*	222	2*mm*	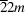	2/*mmm*
	Tetragonal	4	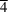	4/*m*	422	4*mm*	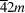	4/*mmm*
	Hexagonal	6	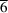	6/*m*	622	6*mm*	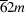	6/*mmm*

**Table:** 

Parity	Motif	Point groups, abstract						
Odd	Monad	*A*_2_	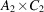		*S*_2_	*S*_2_	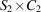	
	Trigonal	*A*_3_	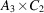		*S*_3_	*S*_3_	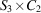	
	Cubic	*A*_4_	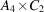		*S*_4_	*S*_4_	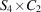	

Even	Dyad	*C*_2_	*C*_2_	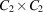	*D*_2_	*D*_2_	*D*_2_	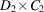
	Tetragonal	*C*_4_	*C*_4_	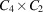	*D*_4_	*D*_4_	*D*_4_	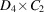
	Hexagonal	*C*_6_	*C*_6_	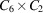	*D*_6_	*D*_6_	*D*_6_	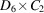

**Table:** (*a*) From *ITA*.

Crystal system	Point groups			
Oblique	1	2		
Rectangular	*m*	2*mm*		
Square	4	4*mm*		
Hexagonal	3	3*m*	6	6*mm*

**Table:** (*b*) This work.

Parity	Motif	Point groups	
Odd	Monad	1	*m*
	Trigonal	3	3*m*

Even	Dyad	2	2*mm*
	Tetragonal	4	4*mm*
	Hexagonal	6	6*mm*

**Table 5:** Summary table of the 18 distinct (non-pairwise isomorphic) groups that arise as 3D crystallographic point groups Those that occur as 2D groups are color-coded red. *ITA* labels are in parentheses. The abstract groups match 1:1 with columns Herman–Mauguin (H-M) odd and even. Schoenflies symbols are for the convenience of readers.

Abstract group	Order	H-M odd	H-M even	Schoenflies symbols
*S*_4_ × *C*_2_	48	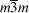		*O*_*h*_
*S*_4_, *S*_4_	24	432, 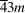		*O*, *T*_*d*_
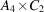	24	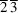 ( 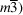		*T*_*h*_
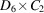	24		6/*mmm*	*D*_6*h*_
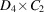	16		4/*mmm*	*D*_4*h*_
*A*_4_	12	23		*T*
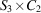 , *D*_6_, *D*_6_, *D*_6_	12	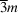	622, 6*mm*, 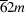	*D*_3*d*_, *D*_6_, *C*_6*v*_, *D*_3*h*_
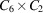	12		6/*m*	*C*_6*h*_
*D*_4_, *D*_4_, *D*_4_	8		422, 4*mm*, 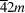	*D*_4_, *C*_4*v*_, *D*_2*h*_
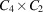	8		4/*m*	*C*_4*h*_
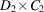	8		2/*mmm* (*mmm*)	*D*_2*h*_
*S*_3_, *S*_3_	6	32, 3*m*		*D*_3_, *C*_3*v*_
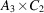 , *C*_6_, *C*_6_	6	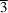	6, 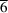	*C*_3*i*_, *C*_6_, *C*_3*h*_
*C*_4_, *C*_4_	4		4, 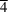	*C*_4_, *S*_4_
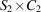 , 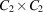 , *D*_2_, *D*_2_	4	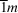 (2/*m*)	2/*m*, 222, 2*mm* (*mm*2)	*C*_2*h*_, *D*_2_, *C*_2*v*_
*A*_3_	3	3		*C*_3_
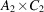 , *S*_2_, *S*_2_, *C*_2_, *C*_2_	2	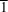 , 2, *m*	2, 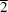 (*m*)	*C*_*i*_, *C*_2_, *C*_*s*_
*A*_2_	1	1		*C*_1_

## Footnotes

1 Henceforth, when we speak of ‘point groups’, we are referring to the point-group classes.

2 *ITA* and Müller (2013 ▸) use the term *family tree* for a Hasse diagram.

3 Here the term *dihedral* is used to identify abstract groups. Crystallographers use the term in the Schoenflies notation. Table 5 includes a comparison.

4 Recall that *S*_*n*_ consists of all *n*! permutations of *n* objects, and its index-2 subgroup *A*_*n*_ consists of all even permutations; see Dummit & Foote (2003 ▸).

5 Table 3.2.1.2 in *ITA* also groups the monoclinics and orthorhombics together.

6 The index of the normalizer of  in  (which is  itself) is the size of its conjugacy class.
